# Mediterranean Diet and Physical Activity: Two Imperative Components in Breast Cancer Prevention

**DOI:** 10.7759/cureus.17306

**Published:** 2021-08-19

**Authors:** Fahmida Dilnaz, Farzina Zafar, Tanzina Afroze, Ummul B Zakia, Tutul Chowdhury, Sanzida S Swarna, Sawsan Fathma, Ruhina Tasmin, Md Sakibuzzaman, Tasnuva T Fariza, Shayet Hossain Eshan

**Affiliations:** 1 Internal Medicine, Jalalabad Ragib-Rabeya Medical College & Hospital, Sylhet, BGD; 2 Pediatric Emergency Medicine, Children's Healthcare of Atlanta, Decatur, USA; 3 Pediatric Emergency Medicine, Emory University School of Medicine, Decatur, USA; 4 Division of Cardiology, University of Washington, Seattle, USA; 5 Internal Medicine, Sir Salimullah Medical College, Dhaka, BGD; 6 Internal Medicine, One Brooklyn Health System, Brooklyn, USA; 7 Addiction Medicine, US Department of Veterans Affairs, Palo Alto, USA; 8 Anesthesiology, Mayo Clinic, Rochester, USA; 9 Internal Medicine, Bangladesh Medical College and Hospital, Dhaka, BGD; 10 Public Health Sciences, University of Southern California Keck School of Medicine, Los Angeles, USA; 11 Internal Medicine, University of Mississippi Medical Center, Jackson, USA; 12 Experimental Pathology (Cancer Biology), Mayo Clinic, Rochester, USA; 13 Neuroscience, California Institute of Behavioral Neurosciences & Psychology, Fairfield, USA; 14 Internal Medicine, Merit Health Wesley, Hattiesburg, USA; 15 Internal Medicine, AMITA Health Saint Joseph Hospital Chicago, Chicago, USA

**Keywords:** breast cancer, mediterranean diet, physical activity, gut microbiome, cancer prevention

## Abstract

Despite tremendous advances in medicine over the past few decades and significantly improved understanding of the symptomology and contributors to breast cancer (BC) incidence, BC rates continue to rise worldwide, with BC being a leading cause of cancer-related death among women. To reduce BC incidence, it is necessary to focus on promoting prevention strategies through a population-based approach of lowering exposure to modifiable risk factors in addition to the application of newer drug interventions (chemoprevention) for prevention in high-risk populations. Currently, available data suggest that lifestyle modifications through a healthy diet and increased physical activity (PA) play a crucial role in BC prevention; specifically, there is growing evidence to indicate that the Mediterranean diet (MeD) lowers cancer risk. This review summarizes the potential role of the MeD and PA in reducing BC risk, with an additional focus on microbial modulation in BC prevention, based on the current evidence obtained from PubMed. After reviewing the immunomodulatory and anticarcinogenic effects of both the MeD and PA, we conclude that further evaluation and proper implementation of both interventions can significantly reduce the risk of BC and associated mortality in the general population.

## Introduction and background

The incidence rate of breast cancer (BC) has been steadily rising by 0.5% each year, and the five-year survival rate for women diagnosed with BC is approximately 90% [1]. Genetic makeup, lifestyle, nutrition, and environmental factors play a significant role in the pathogenesis of BC [2]. Diet and physical activity (PA) are two modifiable lifestyle components that can help reduce BC incidence and hence mortality. The Mediterranean diet (MeD) is widely considered one of the healthiest diets as it mainly comprises natural ingredients that are thought to minimize oxidative stress and inflammation. Researchers postulated that the MeD is linked with changes in the gut microbiome that can have anticancer effects at the cellular level [3]. Women who adhere to the MeD have been shown to have a lower incidence of all subtypes of BC [3]. In contrast, the standard, fat-rich Western diet may increase the risk of estrogen and progesterone-positive BC [4]. A patient’s level of PA appears to be another significant factor in the pathogenesis of BC as it affects various body regulatory systems, including inflammatory mediators, sex hormones, metabolic hormones, adipokines, and gut microbiota [5]. An increasing level of PA has been shown to have an inverse effect on the development of BC [6]. In this paper, we discuss the protective role of the MeD and PA against BC and the effects of both the MeD and PA on microbial composition and function, which could aid in BC prevention. For this study, we reviewed approximately 500 articles, including systematic reviews and meta-analyses, by searching the PubMed database using the terms “Breast cancer prevention,” “Mediterranean diet and breast cancer,” “Physical activity and breast cancer,” and “Microbiome and breast cancer.” In total, 78 papers published over the last five years were included in this review.

## Review

Mediterranean diet in breast cancer prevention

The MeD originated in Italy and Greece in the 1960s [7] and describes the dietary patterns of countries and cultures bordering the Mediterranean Sea. The diet is abundant in plant-based foods rich in complex carbohydrates, such as whole grains, legumes, nuts, beans, vegetables, fruits, herbs, and spices; emphasizes fish, poultry, and lean sources of protein over red and processed meat; uses healthy fat (olive oil) as the principal source of fat; and includes a moderate intake of low-fat dairy and alcohol (mostly red wine) with meals [8].

The MeD is considered one of the healthiest diets because of its focus on plant-based foods rich in antioxidants, fibers (complex carbohydrates), monounsaturated and polyunsaturated fatty acids (PUFAs) (e.g., omega-3), and various phytochemicals, the consumption of which is associated with lower oxidative stress, reduced postprandial glucose variation by increasing insulin sensitivity, and reduced pro-inflammatory cytokines secretion, which reduces inflammation [3,9]. In comparison, the Western diet (WeD) is known to comprise high amounts of refined sugar, red and processed meats, and saturated and transfats, the consumption of which is associated with increased oxidative stress, extreme blood glucose variation, and increased production of pro-inflammatory cytokines [3].

Adherence to the MeD has been shown to protect against diabetes, cardiovascular disease, and some cancers, including lung cancer, colorectal cancer, stomach cancer, prostate cancer, and BC. The largest intervention trial to assess the effect of the MeD on important health outcomes was the Prevención con Dieta Mediterránea (PREDIMED) trial, a multicenter, randomized, controlled, nutritional intervention trial conducted in Spain. The trial considered BC incidence among women as the secondary outcome. The risk reduction of BC was found to be 68% (87-21%) for the MeD with extra virgin olive oil (EVOO) group compared to the control diet group rich in low-fat foods [10]. Several other studies, including clinical trials and observational studies, have also demonstrated an inverse association between the MeD and BC [8,11-14].

A case-control study conducted in Italy and Switzerland in 2018 reported that adherence to the MeD is associated with a reduced risk of BC [13]. Adherence to the MeD was measured through a Mediterranean Diet Score (MDS) ranging from 0 to 9. Compared to an MDS of 0-3, BC risk was reduced with an MDS of 4-5 (odds ratio [OR] = 0.86, 95% confidence interval [CI] = 0.76-0.98), and was further reduced with an MDS of 6-9 (OR = 0.82, 95% CI = 0.71-0.95; p for trend = 0.008), with similar results observed in pre and postmenopausal women [13].

Beneficial Effects of the Key Nutrients of the Mediterranean Diet

Olive oil: Olive oil is a crucial component and a significant source of fat in the traditional MeD. It is rich in monounsaturated fatty acids and polyphenolic compounds. Daily consumption of olive oil is associated with the reduction of certain cancers, including BC. In the PREDIMED trial, high consumption of EVOO was associated with a 62% lower risk of BC compared to the control group (95% CI = 0.16-0.91) [15].

In a systematic review, Buckland and Gonzalez [16] pooled data from six case-controlled studies and found a 38% reduction in BC among those who consumed the highest amount of olive oil. The effects of olive oil components (hydroxytyrosol/oleuropein, oleanolic acid, oleic acid, oleocanthal, and pinoresinol) on triple-negative breast cancer (TNBC) cells have been well-studied [8]. Hydroxytyrosol, a simple phenol found in olive oil, is antitumorigenic and protects against oxidative DNA damage in nonmalignant breast cells in vitro [17]. Oleanolic acid, a triterpenoid, has antiproliferative and antimigratory potential in TNBC cells [18]. Oleic acid, a free fatty acid in olive oil, has a protumorigenic effect [19]. Oleocanthal inhibits the proliferation, migration, and invasion of TNBC cells in vitro [20]. Pinoresinol is considered to be a phytoestrogen due to its similar chemical structure to estrogen and antiproliferative effects on in-vitro BC cells [21].

Whole grains, legumes, and nuts: The MeD is packed with whole-grains such as millet, oats, barley, polenta, brown rice, wheat berries, bread, couscous, and pasta. In several epidemiological studies, the incidence of BC has been found to be lower among women who consume whole grains [22]. Whole grains contain bioactive phytochemicals such as phenolic acids, alkylresorcinols, lignans, vitamin E, polysaccharides, carotenoids, phytosterols, and anthocyanins, which play important roles in the prevention of breast carcinogenesis [22]. An in-vitro study [23] found that germinated wheat flour induces apoptosis and inhibits the growth of human breast cell lines, ultimately preventing cancer cell proliferation. Young barley and its methanolic extract have been shown to prevent the growth of human BC cells by upregulating apoptosis, suppressing cellular proliferation, and arresting the S phase of the cell cycle [24]. Total phenolic extracts in millet inhibit the proliferation of BC cells [25]. Avenanthramide-C in oats prevents the proliferation of human cancer cells by activating apoptosis and caspase activity and by arresting the cell cycle at the G1 phase [26].

Legumes, or “pulses,” like lentils, peas, beans, and chickpeas, are a reliable and affordable source of protein in the traditional MeD. Sangaramoorthy et al. [27] showed that high dietary consumption of beans and other fiber-rich foods lowers estrogen/progestrone receptor-negative BC risk. Other clinical trials [28,29] showed that dietary walnut consumption altered gene expression related to tumor growth, which, in turn, reduced cancer growth and BC risk.

Fruits and vegetables: The MeD is rich in different types of fruits and vegetables, including broccoli, sprouts, cabbage, artichokes, cucumber, beets, eggplant, mushroom, apple, banana, and different nuts. In a study on the association between higher fruit intake during adolescence and early adulthood and the risk of BC, Farvid et al. [30] found an inverse correlation in which increased fruit consumption led to lower rates of BC incidence. An 11% reduced risk of breast cancer was observed among women who consumed ≥5.5 servings/day of fruits and vegetables compared to women who consumed <2.5 servings/day. Constituents of these foods, such as antioxidants and other micronutrients (minerals, phytosterols, polyphenols, sulfides, vitamins, salicylates, glucosinolates, phytoestrogens, lectins, etc.) are essential in reducing the risk of BC as they have antioxidant properties and can prevent activation of carcinogens, suppress spontaneous mutation, and prevent DNA damage [31]. Leafy vegetables contain antioxidants (lutein, zeaxanthin, folates, vitamin A, and carotenoids) that regulate estrogen metabolism and inhibit tumor growth [32]. Fruits, especially red fruits containing ellagic acid, quercetin, and anthocyanins, have anticarcinogenic benefits due to their antioxidant properties. Some polysaccharides found in mushrooms have antitumor and immunomodulatory properties and can help prevent tumor recurrence [33]. However, a meta-analysis of eight major prospective cohort studies found no substantial link between fruit and vegetable consumption and BC risk [34].

Fish and seafood: The MeD recommends moderate consumption of fish and seafood. Evidence regarding the protective effect of fish consumption on BC is inconsistent as some studies have reported a statistically significant inverse association while others have not. A meta-analysis by Laudisio et al. [35] on the Asian population found a statistically significant protective effect of fish consumption against BC (OR = 0.80, 95% CI = 0.73-0.87; p < 0.00001).

The protective effect of fish oil is primarily due to its high content of eicosapentaenoic acid (EPA) and docosahexaenoic acid (DHA) [35,36]. These are long-chain omega-3 PUFAs. One of the key mechanisms by which PUFA halts BC development is by inhibiting the synthesis of arachidonic metabolites, thus minimizing the inflammatory response. EPA limits prostaglandin E2 synthesis by suppressing cyclooxygenase 2 expression. In addition, EPA and DHA downregulate human epidermal growth factor 2 and nuclear factor-kappa light chain enhancer of activated B-cell signaling, thus preventing cell proliferation in BC [37,36]. Finally, EPA and DHA may exert an antitumor effect by regulating gene expression (especially oncogenes and tumor suppressor genes). For example, in an in-vitro study, Shaikh et al. [37] showed that human breast tissue treated with omega-3 PUFA (EPA and DHA) increased the expression of BC suppressor genes, *BRCA1 *and *BRCA2*.

Red meat and processed meat: Although red meat is a major source of protein, increased daily consumption of red meat and processed meat is associated with a high risk of developing BC. The International Agency for Research on Cancer categorized red meat (unprocessed) consumption as “probably carcinogenic for humans” and processed meat consumption as “carcinogenic for humans” [38]. A recent systematic review and meta-analysis linked processed meat consumption with a 9% increased risk of BC. In comparison, unprocessed red meat consumption was associated with a 6% increased risk of BC [39]. Furthermore, a cohort study and meta-analysis conducted in the UK found an increased risk of BC with processed meat consumption, especially in postmenopausal women [40].

There are several possible underlying mechanisms linking red meat intake with BC. Processed meat or red meat cooked at high temperatures is a source of carcinogens such as heterocyclic amines and polycyclic aromatic hydrocarbons [35,41,42]. In addition, high cholesterol content, saturated fat, and heme iron contribute to the malignant potential of red meat and processed meat [39]. The MeD emphasizes a limited intake of red and processed meat, which can be helpful in preventing BC.

Dairy products: A moderate intake of dairy products is recommended in the MeD. Evidence of an association between dairy consumption and breast cancer risk is inconsistent across studies [43-45]. A retrospective cohort study reported an increased risk of developing BC with increased intake of dairy products, especially milk [44]; however, in their meta-analysis, Chen et al. [45] did not find any statistically significant association between dairy product intake and BC. While vitamin D, calcium, and linoleic acid present in dairy may have a protective effect against BC, high saturated fatty acid and endogenous insulin-like growth factor (IGF)-1 may increase the risk [42]. As a result of the heterogeneous composition of dairy products, its net effect on BC prevention remains uncertain.

Microbial Alteration With the Mediterranean Diet

Gut microbiota comprises several microorganisms, including bacteria, yeast, and viruses, that colonize the intestine early on [46]. This composition is shaped by different life events, such that each individual develops a unique microbiota profile. The predominant gut bacterial phyla (according to the taxonomic classification of bacteria) include Firmicutes (consisting of >200 genera such as *Bacillus*, *Enterococcus*, *Lactobacillus*, *Clostridium*, and *Ruminococcus*), Bacteroidetes (consisting predominantly of the genera *Bacteroides *and *Prevotella*), and Actinobacteria (less abundant, primarily composed of the genus *Bifidobacterium*) [47]. The composition of gut microbiota is subject to variation due to several environmental factors such as geography, ethnicity, and lifestyle (predominantly diet) [48]; life events such as illness, infection, and antibiotic treatment [49]; and host genetics [50].

Gut microbes perform several beneficial functions, including synthesizing many essential vitamins (e.g., vitamin B12, folate, vitamin K), regulating host nutrient metabolism, maintaining the structural integrity of the gut mucosal barrier, immunomodulation, and protecting against pathogens [51]. A healthy host-microorganism balance is required to perform these functions optimally. Imbalance in the microbiota (dysbiosis) is associated with several intestinal and extraintestinal diseases, including obesity, diabetes, allergies, infections, neurological disorders, chronic inflammatory and autoimmune conditions, and malignancy [47], where inflammation acts as a major driver of illness [52].

With recent advancements in research, host microbiota has emerged as a major player in many organ-specific cancers, including BC. Microbial alterations are associated with many established risk factors of BC, such as obesity, aging, and higher estrogen levels [53]. In addition to modulating inflammation, gut microbes influence the genomic stability of host cells by deregulating different signaling molecules and metabolites such as short-chain fatty acids (SCFAs), secondary bile acids, and biogenic amines, hence promoting carcinogenesis [53,54]. Gut microbes are also associated with BC progression by affecting the metabolic pathways of estrogen. Certain bacterial β-glucuronidase can deconjugate estrogen (excreted into the intestine through bile after conjugation in the liver), increasing its resorption through enterohepatic circulation, and thus increasing the availability of estrogen to breast tissue [55,56].

**Figure 1:**
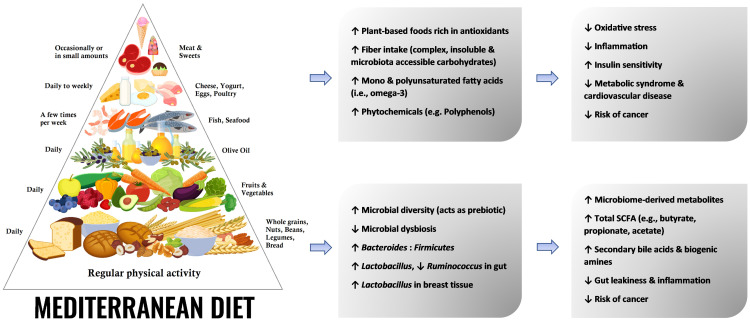
Effects of the MeD on important health outcomes and microbiota. MeD: Mediterranean diet; SCFA: short-chain fatty acid

Further, studies support that probiotics (live bacteria that can restore and maintain healthy microbial composition) and prebiotics (nondigestible fibers that feed the beneficial bacteria in the gut, enhancing their proliferation) have important immunomodulatory and anticarcinogenic effects [53,55,57] and can be used as adjuvants in BC treatment [58]. As the MeD is loaded with complex carbohydrates, it can exert significant prebiotic action [59]. Several studies have demonstrated that a Mediterranean-style diet beneficially modulates the gut microbiome in both humans and experimental animal models, with evidence of increased microbial diversity, higher levels of total SCFAs (such as butyrate, propionate, and acetate) and secondary bile acids [3,60], and increased bacterial-processed bioactive compounds, such as polyphenols (in mammary tissue), that have anticancer properties [61,62] (Figure 1).

Studies comparing MeD and WeD consumers have revealed a higher abundance of *Lactobacillus*, *Clostridium*, *Faecalibacterium*, and *Oscillospira *genera and a lower abundance of *Ruminococcus *and *Coprococcus *genera, as well as a higher Bacteroides:Firmicutes ratio in MeD consumers compared to WeD consumers [63,64]. Additionally, a study involving nonhuman primate models revealed an approximate 10-fold higher abundance of *Lactobacillus *in the mammary gland of monkeys who were fed the MeD and a significantly higher abundance of *Lachnospiraceae*, *Coprococcus*, *Ruminococcus*, and *Oscillospira *in the monkeys who were fed the WeD, suggesting a direct influence of MeD on microbiome population in distal sites other than the intestinal tract, such as the mammary gland [61]. Malignant breast tumors have been found to have distinct microbial populations compared to normal breast tissue, breast skin tissue, and breast tissue in women with benign breast disease, suggesting a possible role of mammary tissue dysbiosis in the development of BC [54,65]. In addition, the use of *Lactobacillus* as a probiotic has been associated with reduced tumor growth, invasion, and metastasis in mouse models, indicating that *Lactobacillus* is a negative regulator of BC [3]. These data suggest that the MeD can significantly influence the microbial population in the gut and mammary tissue, such that microbial modulation through diet can be a promising target in BC prevention and treatment.

Physical activity in breast cancer prevention

PA reduces the risk of BC by regulating multiple essential circulatory mediators such as sex steroid hormones, metabolic hormones, inflammatory mediators, adipokines, and myokines [66,67]. In addition, it modifies the body’s homeostasis by altering plasma metabolites, microRNAs, reactive oxygen species, and exosomes [5,66,67]. Most notably, it adjusts the gut microbiota profile to a beneficial level [5]. A study by Weitzer et al. [68] showed that for both pre and postmenopausal women, physical exercise can have a nearly 20% risk reduction for BC, although postmenopausal women have been found to require high-intensity exercise to derive the same protective effect as premenopausal women [69]. Wu et al. [70] conducted a meta-analysis and found that BC risk can be reduced by 2% for every 25 hours/week of nonoccupational activity, reduced by 3% for every 10 hours/week of exercise, and by 5% for every 2 hours/week of moderate-to-vigorous recreational activity. Weitzer et al. [68] conducted a meta-analysis to determine the importance of the timing of PA and found that morning activity is more protective than afternoon activity, most likely due to the body’s circadian hormonal pattern. Moreover, PA has been found to reduce the risk of primary BC as well as to prevent recurrence and mortality in BC patients [66].

Physical Activity Regulates Sex Steroids and Metabolic Hormones

Women with a high levels of estrogen and androgen have a higher risk of developing BC. A meta-analysis investigating the impact of PA on sex steroids showed that PA diminishes the risk of developing BC by decreasing the level of sex hormones [67]. The beneficial effect of PA is more evident in postmenopausal women as adipose tissue is their primary source of estrogen. A population-based, case-control study found that the timing of PA can also have a protective effect on reducing BC incidence [68]. Morning activity can help reduce the estradiol level shortly after the estradiol peak, around 7:00 am [71]. As estrogen level is proportional to BC risk, the timing of PA certainly has the potential to lower BC risk.

Hyperinsulinemia and insulin resistance can raise the risk of BC by causing increased estrogen production [5]. In addition, insulin exhibits mitogenic, proliferative, and antiapoptotic properties by activating mitogen-activated protein kinase, nuclear factor-kappa light chain enhancer of activated B cells, and phosphatidylinositol 3-kinase/AK strain transforming/mammalian target of the rapamycin signaling pathways, thus promoting BC development [66,67]. Furthermore, hyperinsulinemia increases the synthesis of IGF-1, a well-recognized mitogen responsible for carcinogenesis [72,73]. PA can also reduce insulin resistance and fasting blood glucose levels by reducing weight, thus preventing the development and recurrence of BC [5].

Effects of Physical Activity on Inflammatory Cytokines, Adipokines, and Myokines

A distinct feature of cancer pathogenesis is inflammatory events primarily mediated by cytokines, including interleukin (IL)-1, IL-6, tumor necrosis factor-alpha, and C-reactive protein [5]. Genomic and epigenomic alterations and DNA damage are some of the proposed mechanisms by which chronic inflammation can lead to carcinogenesis. Environmental and lifestyle factors, such as obesity, exhibit a strong association with chronic inflammation and BC development [74]. PA minimizes systemic inflammatory responses and can be a point of interference in the development of BC. Although the impact of PA on an otherwise healthy person is anti-inflammatory, its effect on cytokine level is still subject to debate [5].

Major cytokines, such as adiponectin, leptin, and resistin derived from adipocytes, play a crucial role in the pathogenesis of BC. In contrast to the pro-inflammatory effect of leptin, such as the progression, invasion, and migration of BC, adiponectin has an anti-inflammatory effect and is inversely related to adiposity [5,75]. The ratio of serum adiponectin to leptin is the primary factor determining the pathogenesis of BC. PA can play a crucial role in preventing BC by elevating adiponectin and lowering leptin levels [5].

Myokines, such as myostatin, myonectin, irisin, IL-6, are cytokines produced by myocytes of muscle fiber and veritably modulated by physical activity [5]. These small, multifunctional proteins mediate their effects on angiogenesis and cellular proliferation in a paracrine, autocrine, or endocrine manner. Among the various myokines, irisin is very well studied, and its concentration increases with acute exercise [72]. Preclinical studies have found that irisin can hinder BC viability by enhancing caspase and alleviating nuclear factor-kappa β activity [5,74]. Furthermore, in clinical studies, lower levels of irisin have been found in BC patients, including those with metastatic diseases, suggesting a protective role against BC development and progression [76].

Immunomodulation by Physical Activity

To understand how PA prevents BC or decreases tumor growth in the breast, it is important to understand the role of PA in immunity. PA enhances the immune system via three distinct mechanisms: (1) it helps to get rid of the inflammatory mediators that the body generates in unfavorable situations, for example, increased body mass or prolonged illness; (2) it boosts immunity by increasing the number of immune cells in circulation, such as CD4+ and CD8+ T-cells and natural killer cells; and (3) it counteracts immunosuppressors, myeloid-derived suppressor cells, and T regulatory cells [66]. Thus, PA plays an imperative role in boosting immunity against cancer.

Physical Activity Reduces Oxidative Stress

Oxidative stress may contribute to tumorigenesis as well as BC progression. Oxidative stress occurs when there is an imbalance between oxidant molecules and antioxidants. The antioxidants neutralize the reactive oxygen species and reactive nitrogen species [69]. Interestingly, acute exercise increases oxidant molecules. On the other hand, when PA becomes regular or habitual, the body becomes more efficient at removing these oxidative molecules, increasing the antioxidant effect [73]. Thus, regular PA can help prevent the onset and progression of BC.

Physical Activity Modulates Gut Microbial Composition

PA can help maintain the complex integrity of gut microbiomes. Physical inactivity leads to obesity-induced excess estrogen production, inflammation, and gut dysbiosis. Dysbiosis affects the level of circulating estrogens and alters energy metabolism, leading to tumorigenesis [77]. PA can modify the composition and metabolic product of the gut microbiome by inhibiting obesity-induced inflammatory signaling pathways [5]. Additionally, weight loss lowers the level of circulatory estrogen. However, more studies are needed to fully understand the relationship between PA and its effect on the gut microbiome (Table 1).

**Table 1:** Exercise-induced alteration in systemic mediators associated with the outcome of breast cancer [5,66,74-76,78]. BC: breast cancer; PA: physical activity; NF-κβ: nuclear factor-kappa β

Hormones, immune mediators, adipokines, and myokines	Role in BC	Effect of PA	
			Sex steroid hormone	Increases risk	↓	
Insulin and IGF	Increases risk by increasing circulating estrogen	↓	
Adiponectin	Protects against BC	↑	
Leptin	Increases risk	↓	
Irisin	Can impair BC cells viability by ↑ caspase activity and ↓ NF-κβ	↑	
Catecholamines	Lowers the risk of BC by activating tumor suppressor Hippo signaling pathway	↑	
Natural killer cells, Th1 cytokine production, CD4^+^ T cell proliferation	Protects against BC by enhancing antitumor immunity	↑	
Myeloid-derived suppressor cells, regulatory T-cells (Tregs)	Increases BC risk due to immunosuppressive effects	↓	

## Conclusions

The American Cancer Society recommends minimizing lifetime weight gain through regular exercise and caloric restriction as effective preventive measures against BC. There is substantial evidence showing that PA can lower the risk of BC due to its long-term regulatory effect on various circulatory mediators, such as inflammatory mediators, metabolic hormones, and sex hormones. The MeD is a nutritious diet that aids in maintaining healthy body weight and has a protective effect against BC. Several physiological mechanisms help explain the overall protective effect of the MeD. The consumption of plant-based foods rich in antioxidants and anti-inflammatory properties combined with high PUFA is associated with a reduced risk of BC. Moreover, there is emerging evidence that both the MeD and PA can modulate the gut microbial composition that can assist in BC risk reduction. We strongly believe that a thorough depiction of the protective effects of PA and the MeD in this review will aid in the implementation of these interventions as effective nonpharmacological approaches for the primary prevention of BC.

## References

[1] Siegel RL, Miller KD, Fuchs HE, Jemal A (2021). Cancer statistics, 2021. CA Cancer J Clin.

[2] Iacoviello L, Bonaccio M, de Gaetano G, Donati MB (2021). Epidemiology of breast cancer, a paradigm of the "common soil" hypothesis. Semin Cancer Biol.

[3] Newman TM, Vitolins MZ, Cook KL (2019). From the table to the tumor: the role of Mediterranean and western dietary patterns in shifting microbial-mediated signaling to impact breast cancer risk. Nutrients.

[4] Buja A, Pierbon M, Lago L, Grotto G, Baldo V (2020). Breast cancer primary prevention and diet: an umbrella review. Int J Environ Res Public Health.

[5] Hong BS, Lee KP (2020). A systematic review of the biological mechanisms linking physical activity and breast cancer. Phys Act Nutr.

[6] Moore SC, Lee IM, Weiderpass E (2016). Association of leisure-time physical activity with risk of 26 types of cancer in 1.44 million adults. JAMA Intern Med.

[7] Davis C, Bryan J, Hodgson J, Murphy K (2015). Definition of the Mediterranean diet; a literature review. Nutrients.

[8] Donovan MG, Selmin OI, Stillwater BJ, Neumayer LA, Romagnolo DF (2020). Do olive and fish oils of the Mediterranean diet have a role in triple negative breast cancer prevention and therapy? An exploration of evidence in cells and animal models. Front Nutr.

[9] Schwingshackl L, Morze J, Hoffmann G (2020). Mediterranean diet and health status: active ingredients and pharmacological mechanisms. Br J Pharmacol.

[10] Kargin D, Tomaino L, Serra-Majem L (2019). Experimental outcomes of the Mediterranean diet: lessons learned from the Predimed randomized controlled trial. Nutrients.

[11] van den Brandt PA, Schulpen M (2017). Mediterranean diet adherence and risk of postmenopausal breast cancer: results of a cohort study and meta-analysis. Int J Cancer.

[12] Castelló A, Boldo E, Pérez-Gómez B (2017). Adherence to the Western, Prudent and Mediterranean dietary patterns and breast cancer risk: MCC-Spain study. Maturitas.

[13] Turati F, Carioli G, Bravi F (2018). Mediterranean diet and breast cancer risk. Nutrients.

[14] Castelló A, Ascunce N, Salas-Trejo D (2016). Association between Western and Mediterranean dietary patterns and mammographic density. Obstet Gynecol.

[15] Toledo E, Salas-Salvadó J, Donat-Vargas C (2015). Mediterranean diet and invasive breast cancer risk among women at high cardiovascular risk in the PREDIMED trial: a randomized clinical trial. JAMA Intern Med.

[16] Buckland G, Gonzalez CA (2015). The role of olive oil in disease prevention: a focus on the recent epidemiological evidence from cohort studies and dietary intervention trials. Br J Nutr.

[17] Cruz-Lozano M, González-González A, Marchal JA (2019). Hydroxytyrosol inhibits cancer stem cells and the metastatic capacity of triple-negative breast cancer cell lines by the simultaneous targeting of epithelial-to-mesenchymal transition, Wnt/β-catenin and TGFβ signaling pathways. Eur J Nutr.

[18] Sánchez-Quesada C, López-Biedma A, Gaforio JJ (2015). Oleanolic acid, a compound present in grapes and olives, protects against genotoxicity in human mammary epithelial cells. Molecules.

[19] Marcial-Medina C, Ordoñez-Moreno A, Gonzalez-Reyes C, Cortes-Reynosa P, Perez Salazar E (2019). Oleic acid induces migration through a FFAR1/4, EGFR and AKT-dependent pathway in breast cancer cells. Endocr Connect.

[20] Siddique AB, Ayoub NM, Tajmim A, Meyer SA, Hill RA, El Sayed KA (2019). (-)-Oleocanthal prevents breast cancer locoregional recurrence after primary tumor surgical excision and neoadjuvant targeted therapy in orthotopic nude mouse models. Cancers (Basel).

[21] López-Biedma A, Sánchez-Quesada C, Beltrán G, Delgado-Rodríguez M, Gaforio JJ (2016). Phytoestrogen (+)-pinoresinol exerts antitumor activity in breast cancer cells with different oestrogen receptor statuses. BMC Complement Altern Med.

[22] Xie M, Liu J, Tsao R, Wang Z, Sun B, Wang J (2019). Whole grain consumption for the prevention and treatment of breast cancer. Nutrients.

[23] Cho K, Lee CW, Ohm JB (2016). In vitro study on effect of germinated wheat on human breast cancer cells. Cereal Chem.

[24] Kubatka P, Kello M, Kajo K (2016). Young barley indicates antitumor effects in experimental breast cancer in vivo and in vitro. Nutr Cancer.

[25] Zhang LZ, Liu RH (2015). Phenolic and carotenoid profiles and antiproliferative activity of foxtail millet. Food Chem.

[26] Hastings J, Kenealey J (2017). Avenanthramide-C reduces the viability of MDA-MB-231 breast cancer cells through an apoptotic mechanism. Cancer Cell Int.

[27] Sangaramoorthy M, Koo J, John EM (2018). Intake of bean fiber, beans, and grains and reduced risk of hormone receptor-negative breast cancer: the San Francisco Bay Area Breast Cancer Study. Cancer Med.

[28] Hardman WE, Primerano DA, Legenza MT, Morgan J, Fan J, Denvir J (2019). Dietary walnut altered gene expressions related to tumor growth, survival, and metastasis in breast cancer patients: a pilot clinical trial. Nutr Res.

[29] Elaine Hardman W, Primerano DA, Legenza MT, Morgan J, Fan J, Denvir J (2019). mRNA expression data in breast cancers before and after consumption of walnut by women. Data Brief.

[30] Farvid MS, Chen WY, Michels KB, Cho E, Willett WC, Eliassen AH (2016). Fruit and vegetable consumption in adolescence and early adulthood and risk of breast cancer: population based cohort study. BMJ.

[31] Masala G, Bendinelli B, Assedi M (2017). Up to one-third of breast cancer cases in post-menopausal Mediterranean women might be avoided by modifying lifestyle habits: the EPIC Italy study. Breast Cancer Res Treat.

[32] Farvid MS, Holmes MD, Chen WY, Rosner BA, Tamimi RM, Willett WC, Eliassen AH (2020). Postdiagnostic fruit and vegetable consumption and breast cancer survival: prospective analyses in the nurses' health studies. Cancer Res.

[33] Meng X, Liang H, Luo L (2016). Antitumor polysaccharides from mushrooms: a review on the structural characteristics, antitumor mechanisms and immunomodulating activities. Carbohydr Res.

[34] Hou R, Wei J, Hu Y, Zhang X, Sun X, Chandrasekar EK, Voruganti VS (2019). Healthy dietary patterns and risk and survival of breast cancer: a meta-analysis of cohort studies. Cancer Causes Control.

[35] Laudisio D, Barrea L, Muscogiuri G, Annunziata G, Colao A, Savastano S (2020). Breast cancer prevention in premenopausal women: role of the Mediterranean diet and its components. Nutr Res Rev.

[36] Nindrea RD, Aryandono T, Lazuardi L, Dwiprahasto I (2019). Protective effect of omega-3 fatty acids in fish consumption against breast cancer in Asian patients: a meta-analysis. Asian Pac J Cancer Prev.

[37] Shaikh AA, Braakhuis AJ, Bishop KS (2019). The Mediterranean diet and breast cancer: a personalised approach. Healthcare (Basel).

[38] Krusinska B, Hawrysz I, Wadolowska L, Slowinska MA, Biernacki M, Czerwinska A, Golota JJ (2018). Associations of Mediterranean diet and a posteriori derived dietary patterns with breast and lung cancer risk: a case-control study. Nutrients.

[39] Farvid MS, Stern MC, Norat T (2018). Consumption of red and processed meat and breast cancer incidence: a systematic review and meta-analysis of prospective studies. Int J Cancer.

[40] Anderson JJ, Darwis ND, Mackay DF (2018). Red and processed meat consumption and breast cancer: UK Biobank cohort study and meta-analysis. Eur J Cancer.

[41] Diallo A, Deschasaux M, Latino-Martel P (2018). Red and processed meat intake and cancer risk: results from the prospective NutriNet-Santé cohort study. Int J Cancer.

[42] De Cicco P, Catani MV, Gasperi V, Sibilano M, Quaglietta M, Savini I (2019). Nutrition and breast cancer: a literature review on prevention, treatment and recurrence. Nutrients.

[43] Farvid MS, Eliassen AH, Cho E, Chen WY, Willett WC (2018). Dairy consumption in adolescence and early adulthood and risk of breast cancer. Cancer Epidemiol Biomarkers Prev.

[44] Fraser GE, Jaceldo-Siegl K, Orlich M, Mashchak A, Sirirat R, Knutsen S (2020). Dairy, soy, and risk of breast cancer: those confounded milks. Int J Epidemiol.

[45] Chen L, Li M, Li H (2019). Milk and yogurt intake and breast cancer risk: a meta-analysis. Medicine (Baltimore).

[46] Rodríguez JM, Murphy K, Stanton C (2015). The composition of the gut microbiota throughout life, with an emphasis on early life. Microb Ecol Health Dis.

[47] Rinninella E, Raoul P, Cintoni M, Franceschi F, Miggiano GA, Gasbarrini A, Mele MC (2019). What is the healthy gut microbiota composition? A changing ecosystem across age, environment, diet, and diseases. Microorganisms.

[48] Donovan SM (2017). Introduction to the special focus issue on the impact of diet on gut microbiota composition and function and future opportunities for nutritional modulation of the gut microbiome to improve human health. Gut Microbes.

[49] Iizumi T, Battaglia T, Ruiz V, Perez Perez GI (2017). Gut microbiome and antibiotics. Arch Med Res.

[50] Hughes DA, Bacigalupe R, Wang J (2020). Genome-wide associations of human gut microbiome variation and implications for causal inference analyses. Nat Microbiol.

[51] Thursby E, Juge N (2017). Introduction to the human gut microbiota. Biochem J.

[52] Al Bander Z, Nitert MD, Mousa A, Naderpoor N (2020). The gut microbiota and inflammation: an overview. Int J Environ Res Public Health.

[53] Parida S, Sharma D (2020). Microbial alterations and risk factors of breast cancer: connections and mechanistic insights. Cells.

[54] Mikó E, Kovács T, Sebő É (2019). Microbiome-microbial metabolome-cancer cell interactions in breast cancer-familiar, but unexplored. Cells.

[55] Laborda-Illanes A, Sanchez-Alcoholado L, Dominguez-Recio ME (2020). Breast and gut microbiota action mechanisms in breast cancer pathogenesis and treatment. Cancers (Basel).

[56] Parida S, Sharma D (2019). The microbiome-estrogen connection and breast cancer risk. Cells.

[57] Bultman SJ (2016). The microbiome and its potential as a cancer preventive intervention. Semin Oncol.

[58] Ranjbar S, Seyednejad SA, Azimi H, Rezaeizadeh H, Rahimi R (2019). Emerging roles of probiotics in prevention and treatment of breast cancer: a comprehensive review of their therapeutic potential. Nutr Cancer.

[59] Merra G, Noce A, Marrone G, Cintoni M, Tarsitano MG, Capacci A, De Lorenzo A (2020). Influence of Mediterranean diet on human gut microbiota. Nutrients.

[60] Garcia-Mantrana I, Selma-Royo M, Alcantara C, Collado MC (2018). Shifts on gut microbiota associated to Mediterranean diet adherence and specific dietary intakes on general adult population. Front Microbiol.

[61] Shively CA, Register TC, Appt SE (2018). Consumption of Mediterranean versus Western diet leads to distinct mammary gland microbiome populations. Cell Rep.

[62] Zhou Y, Zheng J, Li Y, Xu DP, Li S, Chen YM, Li HB (2016). Natural polyphenols for prevention and treatment of cancer. Nutrients.

[63] Nagpal R, Shively CA, Appt SA, Register TC, Michalson KT, Vitolins MZ, Yadav H (2018). Gut microbiome composition in non-human primates consuming a Western or Mediterranean diet. Front Nutr.

[64] Krznarić Ž, Vranešić Bender D, Meštrović T (2019). The Mediterranean diet and its association with selected gut bacteria. Curr Opin Clin Nutr Metab Care.

[65] Hieken TJ, Chen J, Hoskin TL (2016). The microbiome of aseptically collected human breast tissue in benign and malignant disease. Sci Rep.

[66] Xu Y, Rogers CJ (2020). Physical activity and breast cancer prevention: possible role of immune mediators. Front Nutr.

[67] Ennour-Idrissi K, Maunsell E, Diorio C (2015). Effect of physical activity on sex hormones in women: a systematic review and meta-analysis of randomized controlled trials. Breast Cancer Res.

[68] Weitzer J, Castaño-Vinyals G, Aragonés N (2021). Effect of time of day of recreational and household physical activity on prostate and breast cancer risk (MCC-Spain study). Int J Cancer.

[69] Ortega MA, Fraile-Martínez O, García-Montero C (2020). Physical activity as an imperative support in breast cancer management. Cancers (Basel).

[70] Wu Y, Zhang D, Kang S (2013). Physical activity and risk of breast cancer: a meta-analysis of prospective studies. Breast Cancer Res Treat.

[71] Bao AM, Liu RY, van Someren EJ, Hofman MA, Cao YX, Zhou JN (2003). Diurnal rhythm of free estradiol during the menstrual cycle. Eur J Endocrinol.

[72] Orlandella FM, De Stefano AE, Iervolino PL, Buono P, Soricelli A, Salvatore G (2021). Dissecting the molecular pathways involved in the effects of physical activity on breast cancers cells: a narrative review. Life Sci.

[73] de Boer MC, Wörner EA, Verlaan D, van Leeuwen PA (2017). The mechanisms and effects of physical activity on breast cancer. Clin Breast Cancer.

[74] Kehm RD, McDonald JA, Fenton SE (2020). Inflammatory biomarkers and breast cancer risk: a systematic review of the evidence and future potential for intervention research. Int J Environ Res Public Health.

[75] Christodoulatos GS, Spyrou N, Kadillari J, Psallida S, Dalamaga M (2019). The role of adipokines in breast cancer: current evidence and perspectives. Curr Obes Rep.

[76] Zhang ZP, Zhang XF, Li H (2018). Serum irisin associates with breast cancer to spinal metastasis. Medicine (Baltimore).

[77] Helmink BA, Khan MA, Hermann A, Gopalakrishnan V, Wargo JA (2019). The microbiome, cancer, and cancer therapy. Nat Med.

[78] Molanouri Shamsi M, Chekachak S, Soudi S (2019). Effects of exercise training and supplementation with selenium nanoparticle on T-helper 1 and 2 and cytokine levels in tumor tissue of mice bearing the 4 T1 mammary carcinoma. Nutrition.

